# Retracted publications in pharmacy systematic reviews

**DOI:** 10.5195/jmla.2022.1280

**Published:** 2022-01-01

**Authors:** Sarah Jane Brown, Caitlin J. Bakker, Nicole R. Theis-Mahon

**Affiliations:** 1 sjbrown@umn.edu, Assistant Librarian, Health Sciences Library, University of Minnesota Libraries, Minneapolis, MN; 2 cjbakker@umn.edu, Associate Librarian, University of Minnesota Libraries, Minneapolis, MN; 3 theis025@umn.edu, Associate Librarian, Health Sciences Library, University of Minnesota Libraries, Minneapolis, MN

**Keywords:** retraction of publication as topic, systematic reviews as topic, research, pharmacy, evidence-based pharmacy practice, publishing

## Abstract

**Objective:**

Systematic reviews and other evidence syntheses, the pinnacle of the evidence pyramid, embody comprehensiveness and rigor; however, retracted data are being incorporated into these publications. This study examines the use of retracted publications in the field of pharmacy, describes characteristics of retracted publications cited by systematic reviews, and discusses factors associated with citation likelihood.

**Methods:**

Using data from Retraction Watch, we identified retracted publications in the pharmacy field. We identified all articles citing these retracted publications in Web of Science and Scopus and limited results to systematic reviews. We classified the retraction reason, determined whether the citation occurred before or after retraction, and analyzed factors associated with the likelihood of systematic reviews citing a retracted publication.

**Results:**

Of 1,396 retracted publications, 283 were cited 1,096 times in systematic reviews. Most (65.0%) (712/1096) citations occurred before retraction. Citations were most often to items retracted due to data falsification or manipulation (39.2%), followed by items retracted due to ethical misconduct including plagiarism (30.4%), or concerns about or errors in data or methods (26.2%). Compared to those not cited in systematic reviews, cited items were significantly more likely to be retracted due to data falsification and manipulation, were published in high impact factor journals, and had longer delays between publication and retraction.

**Conclusions:**

Further analysis of systematic reviews citing retracted publications is needed to determine the impact of flawed data. Librarians understand the nuances involved and can advocate for greater transparency around the retraction process and increase awareness of challenges posed by retractions.

## INTRODUCTION

Retracted publications present a complicated problem for systematic reviews. Systematic reviews, meta-analyses, and other evidence syntheses are considered to be the pinnacle of the evidence pyramid as they embody comprehensive literature searching and rigorous methodological quality assessment [1]. Because few health care providers have capacity to search for and read multiple original research articles to address a clinical question, systematic reviews are intended to serve as easily identifiable evidence upon which clinical decisions can be made. This has resulted in an inherent expectation among clinicians that these works can be relied upon consistently. However, despite the structure, rigor, and importance of systematic reviews, flawed research could be incorporated into these publications. Though retracted publications comprise a small segment of the scientific and medical literature, their presence in systematic reviews holds potential to cause harm by influencing patient care and future research [2].

A recent example highlights the potential impact retracted data could have on patient care. The COVID-19 pandemic, the accompanying growth in published COVID-19 research, and the need for a robust evidence base make a timely illustration of the danger fraudulent data pose and the potential for significant and direct patient harm. As of April 19, 2021, Retraction Watch had identified 105 articles on COVID-19 that were retracted for a variety of reasons since the beginning of the COVID-19 pandemic in January 2020 [3]. Several high-profile COVID-19 drug therapy studies were retracted due to suspected data fraud, creating significant implications for patient care and treatment [4]. Although the COVID-19 pandemic may have amplified issues, poor-quality and duplicative research is not unique to the pandemic. While the aforementioned articles were retracted and discredited promptly, this is not a consistent pattern in the scientific literature. Previous research shows that the time between publication and retraction is between 2 and 3.5 years [5–7]. Even for studies that do not result in retraction, methodological issues may remain. Irrelevant questions, poor study design, and biased reporting are known problems, with an estimated 85% of research generating waste [8]. Economic factors and commercial motives in the pharmaceutical industry may also lead to a focus on short-term gains and less rigor [9].

Retracted publications have been documented and analyzed in a variety of biomedical disciplines, including dentistry [10–12], radiation and oncology [13–15], mental health [16], emergency medicine [17], obstetrics and gynecology [18,19], and surgery [20,21]. Previous studies show that a greater proportion of drug therapy articles are retracted for reasons of misconduct and fraud compared with other biomedical studies [22]. One recent cross-sectional study comparing clinical trials data reporting through publications versus ClinicalTrials.gov found that 74% of studies included at least one discrepancy between the publication and the registry [23]. Earlier research found that only 13.4% of clinical trials reported summary results in ClinicalTrials.gov within one year of trial completion, and only 38.3% reported results at any time [24].

This is concerning because these studies constitute the foundational science that higher evidence is built upon, and providers in all health care fields rely on pharmaceutical and drug literature to identify the most appropriate drug therapies for their patients. If flawed data are incorporated into the higher levels of evidence that these providers seek out, there is a potential for risk to patients' health and safety. The broad reach of pharmaceutical literature combined with the greater proportion of instances of scientific misconduct as compared to other health care disciplines leads us to conclude that the influence of retracted publications in pharmacy systematic reviews deserves further scrutiny [22].

Retractions serve an essential function in scientific literature by allowing science to course correct [11,16,25]. They are also the most visible mechanism to indicate scientific misconduct [26]. Publications may be retracted for a variety of reasons that range from administrative publication errors and mistakes to scientific fraud and misconduct; a single paper may also be retracted for more than one reason [6]. The rise in retractions can be attributed to a number of factors, including increased awareness of retractions and their impact, greater adoption of retraction policies, and adherence to publishing and retraction guidelines such as those issued by the Committee on Publication Ethics (COPE) and the International Committee of Medical Journal Editors (ICMJE) [27–29].

Even with guidelines and increased awareness in identifying retracted publications, there are no standard practices for identifying and labeling these publications. In a study of the representation of retracted publications in mental health, records for 144 retracted articles were examined across seven platforms [16]. Almost half (40%) of those records did not indicate that the article had been retracted, and only 10 of the 144 retracted papers were noted as such across all platforms. These inconsistencies are echoed in later research that assessed 150 retracted articles for compliance with ICMJE's seven recommendations for retracting an article [30]. Only 70 of the 150 articles met all ICMJE criteria.

These inconsistencies may contribute to the continued citation of retracted publications [12]. The array of reasons a publication may be retracted also adds complexity to the issue because portions of a retracted study may still hold value. Continued citation is not inherently problematic as citation does not always equate to endorsement [31]. However, previous research shows that retracted publications are consistently cited positively, implying endorsement of the findings [12]. The aim of this study was to explore the presence of retracted publications in systematic reviews in the pharmaceutical and drug therapy literature.

## METHODS

We obtained a list of all retracted publications in the Retraction Watch Database [32] from inception to May 2019, courtesy of the Center for Scientific Integrity. Retraction Watch is a searchable database of retracted publications and retraction notices, as well as a web presence that highlights stories of retracted publications. For this study, we used a subset of these data and limited our dataset to publications within the Retraction Watch subject classifications of pharmacology, toxicology, and drug design. These subject classifications were chosen due to their applicability to pharmaceutical sciences.

We performed a cited reference search of each retracted publication in both Scopus and Web of Science. These citation indices were chosen for their broad disciplinary scope and ability to perform a cited reference search using a known item. A cited reference search for each retracted item was performed in both databases to ensure a complete set of citing publications. Our searches in each database were limited to the document types “article” and “review.” We then created a single EndNote file with the exported results of our cited reference searches for each retracted item, removing any duplicate citations found. After performing this task for each retracted publication, we then uploaded all results to Rayyan [33] for screening to identify systematic and other evidence-based syntheses (hence referred to as systematic reviews) that cited the retracted publications. Articles were independently screened for inclusion by two reviewers in two phases: title and abstract and full-text screening. Disputes were resolved by consensus. Following full-text screening, we performed a final confirmatory screening to ensure all articles met inclusion criteria.

Systematic reviews were included if they met one or more of the following criteria: self-identified as a systematic review, scoping review, rapid review, meta-analysis, or clinical practice guideline; were PRISMA compliant [34]; or included a detailed methods section outlining multiple databases searched and terms used. Items were excluded if they self-identified as a narrative review, self-identified as consensus-based guidelines, or full text was not available in English. Systematic reviews that had been retracted were excluded from our analysis.

For each retracted publication, we assigned a single reason for the retraction. We mapped the reasons for retraction listed by Retraction Watch and to the taxonomy outlined by Bar Ilan and Halevi [35]: (1) administrative error, such as journals erroneously publishing articles twice or publishing nonfinal versions; (2) ethical misconduct, such as plagiarism, peer review fabrication, or authorship disputes; or (3) scientific distortion, which includes data errors, fabrication, and manipulation. Bar Ilan and Halevi's initial classification of scientific distortion included both intentional and unintentional errors. We further subdivided this classification into (3a) scientific distortion due to falsification or manipulation, describing situations of willful manipulation or falsification of data and (3b) scientific distortion concerns or errors, describing cases where there were errors or other controversy in data, but intention to distort was not confirmed. These updated categories added nuance to the scientific distortion category and isolated intentional manipulation of data from other errors or unintentional mistakes. Where papers were retracted for more than one reason, we assigned the reason for retraction that was the most problematic. For example, a publication retracted for both data fabrication as well as an administrative error was classified as scientific distortion.

Journal impact factor (JIF) quartiles from Clarivate's Journal Citation Reports (JCR) were also added to the dataset of the retracted publications and their citing systematic reviews. Quartile rankings compare a journal's JIF to other journals in its JCR subject area and assign a quartile ranking (Q1, Q2, Q3, Q4). For example, a Q1 journal would have an impact factor within the top 25% of the JIF distribution of its JCR subject area [36]. The use of quartile rankings, as opposed to the JIFs themselves, accounts for disciplinary differences in citation and publishing practices.

Finally, we identified whether the citation to the retracted publication occurred prior to or following retraction. Citation occurred after the retraction if the retraction notice had been published for at least six months prior to the publication of the citing article. A six-month window was used to account for the length of time between manuscript submission and publication, as the median time between submission to online publication is estimated to be 125 days [37].

We compared retracted publications that were and were not cited in systematic reviews. Chi-square tests were conducted to examine relationships between reason for retraction, JIF quartiles, and having been cited in a systematic review, and an independent samples t-test was conducted to compare length of time from publication to retraction between the two groups. Chi-square tests were also conducted to examine relationships between reason for retraction, JIF quartiles, and whether the citation occurred before or after retraction. Statistical tests were performed using R version 3.6.0 [38].

## RESULTS

### Retracted publications in the pharmaceutical literature

Of the 1,396 retracted publications identified, 312 retracted publications were cited 32,559 times. The 1,396 publications were most frequently retracted due to ethical misconduct (553/1396, 39.6%), followed by concerns or errors regarding scientific distortion (383/1396, 27.4%) and scientific distortion due to falsification or manipulation (292/1396, 20.9%). Retracted publications were most frequently published in JIF Q1 journals (505/1396, 36.2%), Q2 journals (322/1396, 23.1%), or journals that had not been assigned a JIF (115/1396, 8.2%).

Of the 312 retracted publications that were cited, 283 were cited 1,096 times in systematic reviews. When considering the differences between retracted items that were cited in systematic reviews and those that were not cited in systematic reviews, we found statistically significant differences in their reason for retraction (X^2^(4,1396)=83.46, *p*<.001), JIF quartile (X^2^(4,1396)=28.45, *p*<.001), and timing of retraction (t(365.05)=-10.805, *p*<.001). Retracted publications cited in systematic reviews were associated with retraction due to scientific distortion—falsification or manipulation and having been published in JIF Q1 or Q2 journals. Retracted publications cited in systematic reviews had a significantly longer period between publication and notice of retraction than those not cited (3.2 years versus 7.0 years; Table 1).

**Table 1:** Characteristics of retracted publications in pharmacy

	Not cited in systematic reviews (n=1,113)	Cited in systematic reviews (n=283)	*p* value
Reason for retraction			*p*<;0.001
Scientific distortion—falsification or manipulation (n=292)	181 (16.3%)	111 (39.2%)	
Scientific distortion—concerns or errors (n=383)	309 (27.8%)	74 (26.2%)	
Ethical misconduct (n=553)	467 (42.0%)	86 (30.4%)	
Administrative error (n=53)	47 (4.2%)	6 (2.1%)	
Unknown (n=115)	109 (9.8%)	6 (2.1%)	
Timing			*p*<0.001
Time between publication and retraction (in days)	1167.05 (± 1509.36)	2559.36 (± 2029.70)	
Journal impact factor			*p*<0.001
Q1 (n=505)	373 (33.5%)	132 (46.6%)	
Q2 (n=322)	248 (22.3%)	74 (26.2%)	
Q3 (n=169)	148 (13.3%)	21 (7.4%)	
Q4 (n=122)	104 (9.3%)	18 (6.4%)	
N/A (n=278)	240 (21.6%)	38 (13.4%)	

### Systematic reviews citing retracted publications

Of the 1,096 systematic review citations to retracted publications, 712 occurred prior to retraction of the publication, while 384 occurred after the publication's retraction. Considering citations that occurred prior to retraction and those that occurred following retraction, there was a statistically significant difference in the JIF quartile (X^2^(4,1096)=29.81, *p*<.0001) and the reason that the cited item was retracted (X^2^(4,1096)=64.221, *p*<.0001).

Systematic reviews that cite retracted publications after retraction were more likely to be published in JIF Q1 journals and less likely to be published in journals with no JIF. Publications retracted due to ethical misconduct were associated with citation in systematic reviews after their retraction, while publications retracted due to scientific distortion—falsification or manipulation were associated with being cited in systematic reviews before the retraction (Table 2).

**Table 2:** Characteristics of citations to retracted publications in systematic reviews

	Cited prior to retraction (n=712)	Cited after retraction (n=384)	p-value
JIF of citing systematic review			*p*<0.0001
Q1 (n=482)	275 (38.6%)	207 (53.9%)	
Q2 (n=260)	186 (26.1%)	74 (19.3%)	
Q3 (n=128)	82 (11.5%)	46 (12.0%)	
Q4 (n=65)	44 (6.2%)	21 (5.5%)	
N/A (n=161)	125 (17.6%)	36 (9.4%)	
Reason for retraction of the publication			*p*<0.0001
Scientific distortion—falsification or manipulation (n=431)	321 (45.1%)	110 (28.7%)	
Scientific distortion—concerns or errors (n=427)	285 (40.0%)	142 (37.0%)	
Ethical misconduct (n=217)	100 (14.1%)	117 (30.5%)	
Administrative error (n=12)	4 (0.6%)	8 (2.1%)	
Unknown (n=9)	2 (0.3%)	7 (1.8%)	

## DISCUSSION

Our findings show that retracted publications continue to be cited in systematic reviews in the field of pharmacy. Almost a quarter (20.3%) of the retracted publications in this sample were cited by one or more systematic reviews, and these citations occurred both before and after the issuance of the retraction notice. Because retractions are not consistently or clearly represented across resources [16], it is possible that systematic review authors are unaware that a particular publication has been retracted. Further investigation into the nature of these citations is needed to determine how retracted publications are utilized to support systematic review findings.

Systematic reviews and other evidence-based syntheses occupy the top spot in the evidence hierarchy [1], and librarians teach students to look for systematic reviews to answer clinical questions. Although the reputational and career implications of retraction are recognized by faculty [39], as are the ethical issues of student plagiarism and research misconduct and their implication for the student's academic career [40], the clinical and educational implications of retractions as potential evidence have not been fully addressed in health sciences education. If further analysis of citations to retracted publications showed that systematic reviews regularly incorporate flawed data into their findings and recommendations, what do health sciences students, faculty, and health care providers need to know about retractions and how to incorporate retractions into one's knowledge base? Librarians may be uniquely positioned to play a broader educational role in teaching students about retractions and how retracted data can potentially influence a systematic review. We can teach updated critical appraisal skills and incorporate techniques for assessing the level of rigor applied to a single systematic review. Librarians can also influence the broader awareness of retractions among health sciences students and faculty.

When assessing scientific literature, researchers may use metrics such as the JIF as an indicator of journal quality or rigor [41]. Our investigation found that both the retracted publications and systematic reviews citing the publication after the retraction was issued were more likely to be in JIF Q1. The use of the JIF as a proxy for scientific quality and rigor has previously been questioned [42,43]. Our results support further questioning of this assumption, as well as the assumption that retracted publications are more frequently the problem of less rigorous, less impactful journals, and emphasize the importance of critical appraisal of individual articles.

Critical appraisal involves revisiting previously read materials and integrating new findings into one's understanding. This may be particularly important in the case of fundamentally flawed research, as the retraction process takes time. Fraud and ethical misconduct in scientific studies typically take longer to discover than blatant plagiarism or administrative errors [6], and this is likely because concerns need to be raised and investigations need to be conducted thoroughly [35]. In our sample, retracted publications that were cited in systematic reviews had a significantly longer period between publication and notice of retraction than those that were not cited (3.2 years versus 7.0 years). The lag time between publication and retraction creates more opportunities for retracted data to be used in systematic reviews. This may be less of an issue for reviews that are on a regular update schedule, such as clinical practice guidelines and Cochrane systematic reviews, but does present a problem for standalone systematic reviews.

The need for regular updates of systematic reviews is well established, although challenging in practice. In one study of 100 systematic reviews published between 1995 and 2005, it was found that 23% of reviews were out of date within two years of publication, 15% within one year, and 7% were outdated by the time they were published [44]. While the discussion surrounding the need for updates has largely centered around the emergence of new and contradictory evidence, the inclusion of potentially erroneous data in the initial systematic review heightens the urgency of the need for regular updates. This problem may present an opportunity for librarians to advocate for updates to both systematic review guidance for authors and guidance for publishers to address this problem.

Some systematic review guidance encourages authors to search for and consider retractions [45], while other guidelines do not mention retractions at all [46]. Searching for retractions should be built into the systematic review workflow and can be accomplished through known-item search using resources such as the Retraction Watch Database [32]. However, while procedures to identify retractions among search results may be integrated into the systematic review process, little direction currently exists as to how the retraction should be handled when it comes to inclusion in the systematic review. While it may seem prudent to exclude a retracted publication due to fabrication of data, others have argued that excluding a publication based on its retracted status alone would not align with best practices if that item had met the inclusion and exclusion criteria specified in the systematic review protocol [47]. If systematic review authors can determine the reason for retraction and its potential impact on the systematic review's findings, it allows them to assess the trustworthiness of the data and make decisions about the inclusion of the study. However, retraction notices are often vague, lacking in sufficient detail to determine exactly which parts of the paper may be problematic and the nature of the concerns [48].

The concerning degree of variability in how retractions are represented across databases and a lack of full adherence to COPE and ICMJE guidelines has been well reported [16,30]. Journal retraction policies vary, and retraction notices may list an explicit reason for retraction. Ambiguity and a lack of context or detail may obscure the severity of the problem [10,49,50]. For journal editors, clearer guidance on describing the reason for retraction and better adherence to COPE guidelines may address the opacity and inconsistency in retraction notices. Librarians, as chief negotiators with publishers, can advocate for better transparency around the retraction process and more context and detail in the issued retraction notices.

Literature in the field of pharmacy and the confidentiality around drug development present some unique considerations. Investigations into drug therapy literature have found that the proportion of retractions due to scientific misconduct are significantly higher when compared to the broader biomedical literature [22]. Nearly half of all retracted publications in the field of anesthesia and analgesia are due to fraud or misconduct, although just four authors are responsible for nearly 60% of the retracted anesthesia literature [51]. There is a lack of a culture of sharing and transparency in the pharmaceutical sciences, as drug development data are often proprietary and manufacturers have business interests in keeping it confidential. However, clinical trial data do not fall under the same legal protections as data about product development [52]; studies have shown that data are not reported to ClinicalTrials.gov in a timely manner despite obligations to do so [24,53]. Another study of a sample of 489 ICMJE-affiliated journals found that 56% merely referred to the guidelines without specifying a data-sharing policy [54]. This lack of transparency and explicit guidance may contribute to flawed data entering the peer-reviewed literature and their subsequent citation. One possible solution to assist authors in assessing data, and in preventing the use of flawed data, would be to publish and share data before publication. This increases transparency and reproducibility and has the potential for errors or flaws to be caught before appearing in the literature [49].

This transparency would benefit both information users and information creators, including those whose work has been retracted. There is a stigma around retractions [55]. Retractions imply culpability and have an inherently negative connotation and impact on authors, potentially penalizing honesty. Greater clarity around the reason for retraction and a more nuanced taxonomy for describing reasons for retractions may provide more context and encourage authors to correct unintentional errors. Several researchers have argued for publishers to establish a self-retraction process that would be initiated by the author to correct these types of errors and mistakes and distinguish this type of retraction from one originating from fraud or misconduct [55,56]. This more nuanced approach to retractions, combined with greater transparency in retraction notices and processes, could contribute to enabling users to make informed decisions about when and how they might incorporate retracted articles into their research and practice.

This is not a comprehensive list of retracted publications cited by systematic reviews in the field of pharmacy. The Retraction Watch Database does not currently have subject headings devoted to pharmacy practice. As such, this sample of articles is more representative of basic and pharmaceutical sciences. Pharmacists and those interested in pharmacy practice rely on literature that may not be represented by the titles included in this study since these categories are not necessarily inclusive of social and administrative pharmacy practice. We did not assess the quality of the systematic reviews that were included in our analysis, nor did we assess whether the retracted publications were noted as such in the systematic reviews. As a result, the systematic reviews included in this sample may be of lower quality or may indicate that the publication in question has been retracted.

In conclusion, the presence of retracted publications in pharmacy systematic reviews raises concerns. In this study, 20.3% of retracted publications were cited in systematic reviews. Of these citations, 39.3% of citations were to items retracted due to data falsification or manipulation. This is of particular concern for the field of pharmacy, with implications for human, animal, and environmental health. Further analysis of the systematic reviews citing retracted publications is needed, including an assessment of the nature of the citation and the methodological quality of the systematic review, to understand the scale of the problem and determine the impact of flawed data on systematic review conclusions and recommendations.

## Data Availability

The data underlying this publication are available through the Center for Scientific Integrity's Retraction Watch Database and are subject to the provisions of a Data Use Agreement. Individuals may request access to this data by contacting the Retraction Watch Database at team@retractionwatch.com.
